# On the Wear Mechanism and Subsurface Deformation of Zr-Based Metallic Glass at Subzero Temperature

**DOI:** 10.3390/ma18133012

**Published:** 2025-06-25

**Authors:** Xin Li, Jianan Fu, Zhen Li, Fei Sun, Kaikai Song, Jiang Ma

**Affiliations:** 1 School of Mechanical, Electrical & Information Engineering, Shandong University, Weihai 264209, China; lixin2018@email.szu.edu.cn; 2 State Key Laboratory of Radio Frequency Heterogeneous Integration, College of Mechatronics and Control Engineering, Shenzhen University, Shenzhen 518060, China; 3 Preparation and Application of Aerospace High-Performance Composite Materials, Future Industry Laboratory of Higher Education Institutions in Shandong Province, Shandong University, Weihai 264209, China; 4 Department of Mechanics and Aerospace Engineering, Southern University of Science and Technology, Shenzhen 518055, China; fujn2022@mail.sustech.edu.cn (J.F.); liz33@sustech.edu.cn (Z.L.); 5 Department of Materials Science and Engineering, Fujian University of Technology, Fuzhou 350118, China; sunfei2016@fjut.edu.cn

**Keywords:** metallic glasses, wear properties, subzero temperature, wear mechanism, subsurface deformation

## Abstract

Metallic glasses (MGs) with excellent mechanical properties have significant applications in frontier technological fields such as medical, energy and aerospace industries. Recently, MGs have been considered as ideal candidates for subzero engineering applications due to their disordered atomic structure array. However, the mechanical properties and wear behaviors of MGs at subzero temperatures have rarely been explored. In this work, the wear properties and wear mechanisms of Zr-based MG were systematically evaluated at a subzero temperature of −50 °C. Compared to the wear results at room temperature, MG in a subzero environment shows a ~60% reduction in wear rate. The main contributing factor is that MG at room temperature will easily forms a thin, brittle oxide layer at the sliding interface, which will lead to oxidation, adhesive and abrasive wear on its surface, whereas these wear behaviors do not occur in subzero conditions where only abrasive wear occurs. Meanwhile, MG at subzero temperatures has a higher elastic modulus. These properties make MG more wear-resistant in subzero environments. The current study will provide new perspectives on the wear mechanisms and subsurface deformation of MG in subzero environments and valuable insights into the use of MG in subzero engineering applications, such as deep space and polar exploration.

## 1. Introduction

With the continuous advancement of science and technology, human exploration has progressively extended into unknown and extreme environments such as deep space, polar regions and the deep sea [1,2]. These regions are typically characterized by extremely low temperatures, which differ significantly from the ambient conditions under which conventional equipment is designed to operate [3,4]. Under such harsh environments, the operational stability and reliability of exploration devices are severely challenged, placing stringent demands on their structural materials and critical components [5]. Among various failure mechanisms, wear is one of the primary causes of performance degradation and even catastrophic failure, particularly under long-term operation and complex loading conditions [6,7]. Therefore, investigating the wear behavior of materials in cryogenic environments is essential for enhancing the service life and operational stability of equipment in extreme conditions [8,9]. However, most existing engineering materials are designed for ambient applications [10,11,12]. While they perform well under conventional conditions, they often experience abrupt performance degradation or even sudden failure at low temperatures [13]. The identification or development of novel materials with excellent tribological properties under cryogenic conditions has become a critical requirement to ensure the long-term and stable operation of exploration and storage equipment in extreme environments [14,15].

As a novel class of materials, metallic glass (MG), also known as amorphous alloy, has attracted significant attention due to its unique disordered atomic structure, which endows them with outstanding properties such as corrosion resistance, thermoplasticity, ultrasonic vibration-induced plasticity, soft magnetism and effective catalyst properties [16,17,18,19,20]. These attributes have led to widespread applications in fields including welding, energy storage, energy conversion, fuel cells and micro-electromechanical systems [21,22,23,24,25,26,27]. Among their many remarkable characteristics, the exceptional mechanical properties of MGs are particularly notable. Unlike conventional crystalline materials, MGs exhibit a high elastic strain limit and strength, resulting in superior wear resistance [9,28]. Previous studies have demonstrated excellent wear performance across various MG systems [9,29,30]. However, research on their wear behavior and underlying mechanisms has predominantly focused on room and elevated temperature conditions [30,31,32,33]. Although MGs maintain excellent mechanical properties at low temperatures, such as plasticity and compressive strength, studies on their wear performance and corresponding mechanisms under cryogenic conditions remain limited [34,35]. Advancements in this research field are crucial for facilitating the application of MGs in extremely low-temperature environments.

In this study, a Zr-based MG that possesses a unique combination of high glass forming ability (GFA), mechanical strength and corrosion resistance is employed to investigate its wear behavior at −50 °C, exhibiting a focus on elucidating the underlying wear mechanisms [36]. Compared to room-temperature conditions, the Zr-based MG exhibited a lower wear rate, reduced coefficient of friction (COF) and distinct wear mechanisms at low temperatures. Adhesive wear and oxidative wear are identified as the dominant mechanisms governing its wear performance, as confirmed by subsurface analysis beneath the wear tracks. Moreover, the higher elastic modulus of MG at subzero temperatures contributes to its enhanced wear resistance under subzero temperatures. This study provides meaningful insights into the tribological behavior of MGs at low temperatures and shows potential for expanding their applications in subzero temperature environments such as deep space exploration.

## 2. Materials and Methods

### 2.1. Materials Preparation and Characterization

This study used commercial alloy as the raw material. A Zr-based MG with a composition of Zr_35_Ti_30_Cu_8.25_Be_26.75_, preprocessed via ultrasonic cleaning with ethanol to remove surface contaminants, was fabricated via a copper mold casting process into plates with a thickness of 3 mm and lateral dimensions of 40 mm × 100 mm under a high-purity argon atmosphere. Circular specimens with a diameter of 15 mm were obtained from these plates using AP250L wire electrical discharge machining(Sodick Co., Ltd., Yokohama City, Japan). To minimize the influence of surface roughness on subsequent wear tests, the specimens were polished to a roughness average (Ra) < 50 nm, with the final measured Ra about 3 nm. After polishing, the specimens were ultrasonically cleaned in ethanol and subsequently stored in a vacuum environment prior to testing.

### 2.2. Wear Test in Subzero and Room Temperature

Prior to conducting the wear test at subzero temperatures of −50 °C, the critical wear components were placed inside a chamber capable of achieving subzero conditions. The chamber was then cooled down to the target temperature, after which cooling was halted. The temperature within the chamber was continuously monitored for the following 5 min to ensure temperature stability, with fluctuations maintained within ±0.5 °C. Once stable conditions were confirmed, the wear tests were initiated. The wear performance of the Zr-based MG was evaluated under a contact sliding mode using a conventional ball-on-disk configuration. The tests were performed on an MFT-5000 tribometer (Rtec Instrument, San Jose, CA, USA). To minimize the influence of ambient humidity on the wear behavior, the ambient relative humidity was maintained at 45% throughout the experiment. A bearing steel ball of 6.5 mm in diameter, known for its high hardness (62 HRC), was used as the counter-body to reduce its wear and ensure consistency in results. The testing parameters included a rotary offset radius of 3 mm and a sliding velocity of 200 mm/s. Normal loads of 15 N and 30 N were applied, both representative of typical engineering applications. Each test lasted for 30 min, allowing the Zr-based MG to reach a steady-state wear condition. To enable comparative analysis, wear experiments at room temperature (25 °C) were also conducted under identical test conditions.

### 2.3. Microstructure Characterizations

#### 2.3.1. X-Ray Diffraction (XRD)

Phase analysis was performed on a Rigaku Miniflex 600 diffractometer (Rigaku Corporation, Akishima-shi, Tokyo, Japan) using Cu-Kα radiation (*λ* = 1.5406 Å). Scans were conducted over the 20–90° 2*θ* range with continuous scanning mode at 40 kV/15 mA and a 0.02° step size with a scan rate of 3°/min.

#### 2.3.2. Scanning Electron Microscopy (SEM)

The morphology of the worn surface inside the wear track and that close to the wear track edge was observed using a Quanta 450FEG SEM (Thermo Fisher Scientific Inc., Waltham, MA, USA) operated at an accelerating voltage of 15 kV. The worn surface was also examined using energy-dispersive X-ray spectrometry (EDS) to evaluate possible oxidation.

#### 2.3.3. Transmission Electron Microscopy (TEM)

TEM analysis was conducted using a Titan Cubed Themis G2 300 system (Thermo Fisher Scientific Inc., Waltham, MA, USA) operated at 200 kV. Specimens were prepared via focused ion beam (FIB) milling on a Scios dual-beam system (Thermo Fisher Scientific Inc., Waltham, MA, USA), with Pt-protected regions (5 μm × 1.5 μm × 2 μm), which were thinned to ≤100 nm. High-angle annular dark-field scanning transmission electron microscopy (HAADF-STEM) imaging coupled with EDS elemental mapping revealed interfacial diffusion characteristics. Selected-area electron diffraction (SAED) patterns and high-resolution transmission electron microscopy (HRTEM, equipment in TEM) were used to further investigate the crystallinity and lattice structure of the samples.

#### 2.3.4. Nanoindentation

A Hysitron TI 950 TriboIndenter (Bruker Corporation, Los Angeles, CA, USA) equipped with a Berkovich indenter performed nanoindentation tests. Nanoindentation was selected for its high spatial resolution to characterize localized mechanical properties in wear-affected subsurfaces (<1 µm), enabling direct and precise measurement of hardness and elastic modulus via the Oliver–Pharr method [37]. Experiments used a 4 × 4 indentation array per sample (coefficient of variation < 5%), with a peak force of 50 mN (maximum penetration of about 700 nm, maintaining about < 10% of sample thickness to avoid substrate effects [38]). Surface roughness (Ra = 3.3 nm) was mitigated by ensuring indentation depth exceeded 50× Ra (165 nm), validated via WLI pre-testing to probe bulk properties [38]. Consequently, a peak load of 50 mN was applied to ensure the actual indentation depth exceeded 165 nm, with a 2 s holding time at maximum load to minimize creep effects.

#### 2.3.5. White Light Interferometry (WLI)

Ra of polished samples was ≤50 nm, measured via WLI (Contour GTX, Bruker Corporation, Los Angeles, CA, USA) using a 50× Mirau objective (numerical aperture = 0.55) over 5 random 40 × 40 μm^2^ areas. Post-wear surface topography (3 × 3 mm^2^ scan area centered on wear tracks) was analyzed using the same WLI system, with a step resolution of 0.1 μm. Cross-sectional profiles were extracted from representative homogeneous regions.

#### 2.3.6. Modulus Testing Machine

The elastic modulus was measured over a range of temperatures using an RFDA LT 80 system (IMCE, Leuven, Belgium) equipped with the impulse excitation technique. Zr-based MG plates (40 mm × 12 mm × 3 mm) were cut by electrical discharge machining and suspended in the test chamber on thin metallic wires. The chamber temperature was controlled by a precision temperature module with an accuracy of ±0.5 °C. At each target temperature, the specimen was lightly struck by a ceramic rod, inducing flexural and torsional vibrations. A highly sensitive microphone inside the chamber recorded the acoustic response, which was converted to elastic modulus data via Fourier transformation (FFT) of the resonance frequencies.

## 3. Results and Discussion

### 3.1. Zr-Based MG Microstructure and Properties

**Figure 1:**
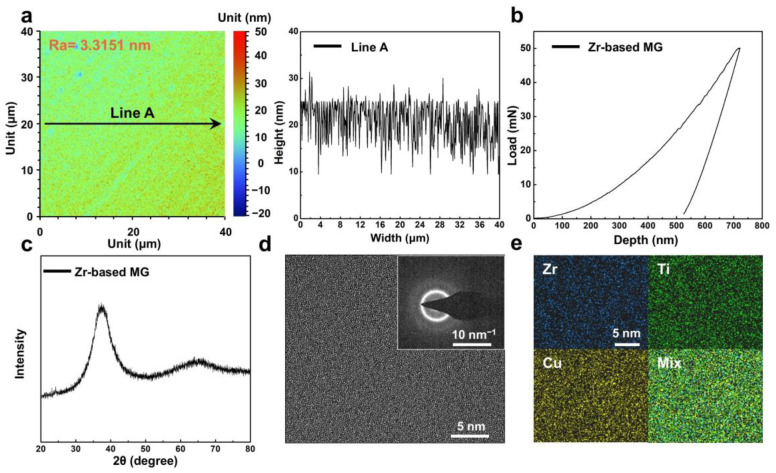
The microstructure of Zr-based metallic glass (MG). (**a**) Roughness distribution map and corresponding linear distribution plot of Zr-based MG surface. (**b**) Typical nanoindentation load–displacement curves of Zr-based MG. (**c**) X-ray diffraction (XRD) pattern of Zr-based MG. (**d**) High-resolution transmission electron microscopy (HRTEM) image and corresponding SAED pattern of Zr-based MG and corresponding energy-dispersive X-ray spectrometry (EDS) mapping are shown in (**e**).

The as-cast Zr-based MG was subjected to comprehensive structural and mechanical characterization. Figure 1a presents the surface roughness of the specimens used for the wear tests. After mechanical polishing, the Ra was reduced to 3.3151 nm, substantially minimizing any interference of surface texture on the wear measurements. Nanoindentation testing revealed that the Zr-based MG exhibited excellent mechanical properties at room temperature, with an average hardness of 5.56 GPa and an elastic modulus of 93.55 GPa (Figure 1b). Figure 1c shows the XRD pattern of the prepared sample, which shows only a broad diffuse diffraction peak and no obvious crystallization peaks, proving its amorphous structure [39]; observation by TEM further verifies its atomic-scale disordered characteristics (Figure 1d). EDS elemental mapping in Figure 1e indicates a uniform distribution of Zr, Ti and Cu throughout the material, with no evidence of elemental segregation.

### 3.2. Zr-Based MG Wear Behavior in Subzero and Room Temperature

**Figure 2:**
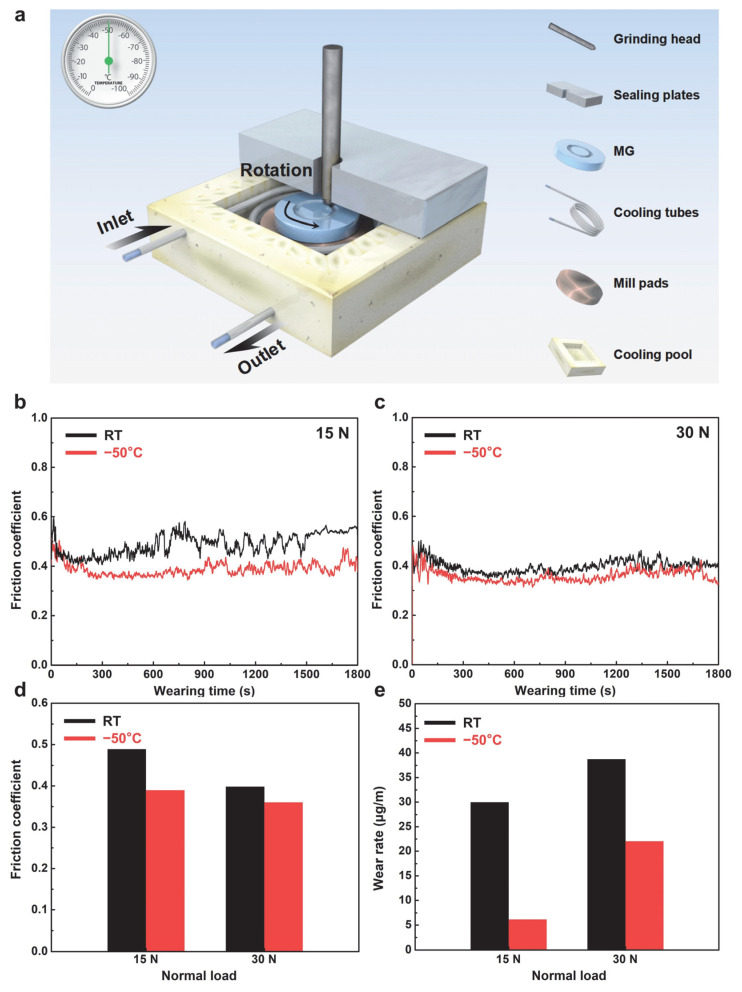
Wear behavior of Zr-based MG in subzero and room temperature. (**a**) Schematic illustration of low-temperature wear test process setup. The real-time evolution of the coefficient of friction (COF) as a function of sliding time under normal loads of (**b**) 15 and (**c**) 30 N. The friction coefficients (**d**) and wear rates (**e**) under the various load and temperature conditions.

**Figure 3:**
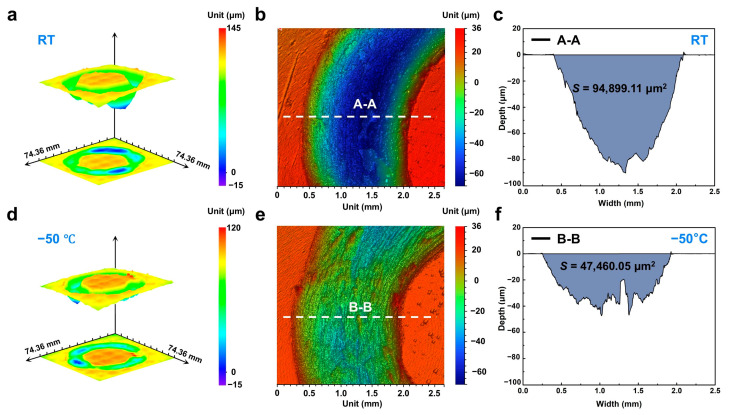
Three-dimensional wear morphology of Zr-based MG. (**a**) Three-dimensional topographies of the wear tracks generated at room temperature; (**b**) magnified view of uniformly worn regions highlighting homogeneous material removal characteristics; (**c**) Cross-sectional profile corresponding to A-A maker in (**b**). (**d**) Three-dimensional topographies of the wear tracks generated at −50 °C; (**e**) magnified view of uniformly worn regions highlighting homogeneous material removal characteristics; (**f**) Cross-sectional profile corresponding to B-B maker in (**e**).

Figure 2a schematically illustrates the wear test setup in subzero temperature. During the experiments, ethanol was circulated through tubing connected to a refrigeration system around the Zr-based MG, and its evaporative cooling effect was used to stabilize the chamber temperature at −50 °C. Figure 2b,c present the real-time evolution of the COF as a function of sliding time under normal loads of 15 N and 30 N, respectively. The COF was calculated as the ratio of the constant normal force to the measured tangential force (COF = *F*_normal force_/*F*_tangential force_). Under all load and temperature conditions, the wear curves exhibit a rapid initial rise followed by a steady-state plateau, corresponding to the transition from the run-in to the steady-state wear regime [40,41]. Notably, the COF at −50 °C remains consistently lower than that at room temperature: under 15 N, the average COF decreases from 0.488 at room temperature to 0.389 at −50 °C; under 30 N, it decreases from 0.398 to 0.359, a difference of approximately 0.039 (Figure 2d). Furthermore, all measured COF values exceed 0.35, indicating pronounced dry friction behavior, and no values approach 0.1 (typical of ice lubrication) [42,43], suggesting that ice lubrication effects on the low-temperature wear response of the Zr-based MG are negligible [44,45]. Figure 2e shows the wear rates under various load and temperature conditions, calculated as the mass loss per sliding distance (Wear rate = *m*_loss mass_/*d*_wear distance_). At 15 N, the room-temperature wear rate (29.96 × 10^−6^ μg/m) is approximately five times higher than at −50 °C. At 30 N, the wear rate increases from 22.04 × 10^−6^ μg/m at −50 °C to 38.70 × 10^−6^ μg/m at room temperature (Figure 3e). These results demonstrate that the Zr-based MG exhibits superior wear resistance at subzero temperatures and that its wear mechanisms differ markedly from those at ambient conditions.

### 3.3. Zr-Based MG Three-Dimensional (3D) Wear Morphology

An optical 3D imaging system was employed to quantitatively characterize the wear scar morphology. Figure 3a,d show the 3D topographies of the wear tracks generated at −50 °C and room temperature, respectively, over a 7 mm × 7 mm scan area centered on the contact region. It is evident that the room-temperature scars are significantly deeper and more heterogeneous, exhibiting pronounced grooves in localized regions, whereas the scars after subzero temperature are overall shallower and display a more uniform profile. To quantitatively assess scar depth, cross-sectional profiles were extracted from representative areas exhibiting uniform wear (Figure 3b,e). Analysis of these profiles reveals a maximum scar depth of approximately 90 μm at room temperature, compared to only about 40 μm under subzero temperature. By integrating the area under these profiles, the wear cross-sectional area at −50 °C was determined to be 47,460.05 μm^2^, substantially lower than the 94,899.11 μm^2^ measured at room temperature (Figure 3c,f). These findings further corroborate the observed reduction in wear rate for the Zr-based MG under subzero temperatures.

### 3.4. Zr-Based MG Wear Mechanism in Subzero and Room Temperature

**Figure 4:**
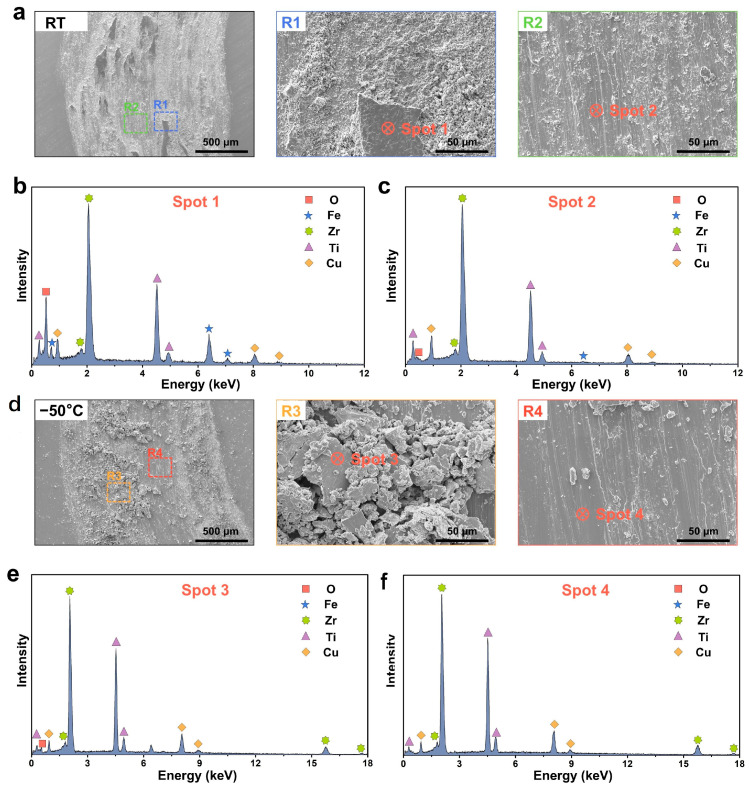
Wear mechanism of Zr-based MG. (**a**) The wear scar microstructure of Zr-based MG and corresponding magnified images at room temperature. And EDS of the wear debris region corresponding to (**b**) Spot 1 and (**c**) Spot 2 in (**a**). (**d**) The wear scar microstructure of Zr-based MG and corresponding magnified images at −50 °C. And EDS of the wear debris region corresponding to (**e**) Spot 3 and (**f**) Spot 4 in (**d**).

To accurately investigate the wear deformation and mechanisms of Zr-based MG under subzero temperature, detailed observations of the wear scar microstructure were conducted. Following wear at room temperature, the wear grooves exhibited an “island-like” morphology, a heterogeneous topology that significantly increased surface roughness and accounted for fluctuations in the COF under a 15 N load. In contrast, at −50 °C, no island-like features were observed; instead, the grooves contained large wear debris fragments approximately 50 μm in diameter. At the same magnification, numerous smaller debris particles (≈5 μm) were also detected around the island-like regions at room temperature. These fine particles, inferred to originate from island-edge fragmentation, suggest that room-temperature debris is more brittle than that formed under subzero temperature. Furthermore, examination of regions devoid of debris revealed that room-temperature samples exhibited markedly more ploughing grooves than samples of subzero temperature, with small particles frequently adhering to the groove edges, whereas the grooves formed at −50 °C remained comparatively clean (Figure 4a,d).

To elucidate the wear mechanisms of the Zr-based MG under room temperature and subzero temperature, elemental analyses were conducted on selected regions (see Table 1 for elemental concentrations). Notably, EDS analysis shows a relative Ti content higher than the nominal composition, likely due to semi-quantitative measurement limitations, stronger X-ray signals from Ti compared to Zr and surface Ti oxide layer formation that enhances Ti detectability. After room-temperature wear, EDS of the wear debris region (Spot 1) revealed a pronounced O peak, accounting for 50.41 at.%, in addition to the base metal elements, indicating the formation of an oxide layer due to localized frictional heating during repeated sliding [46,47]. A significant signal of Fe was also detected at Spot 1 (Figure 4b), demonstrating that material from the counter-body had adhered to the Zr-based MG surface. In contrast, the debris-free region (Spot 2) exhibited only trace O and no detectable Fe (Figure 4c). Together with the observed surface morphology, these results indicate that at room temperature, oxidative wear, adhesive wear and abrasive wear act in concert to wear the Zr-based MG.

Under −50 °C conditions, although instantaneous frictional heating still occurs at the contact interface, the generated heat rapidly dissipates into the cold environment. EDS of the wear debris region at −50 °C (Spot 3) showed only minor O enrichment (25.78 at%) and no Fe signal (Figure 4e), while the debris-free region (Spot 4) exhibited an oxygen concentration of merely 7.7 at. % without any Fe (Figure 4f). These findings demonstrate that under subzero temperature, the dominant wear mechanism of the Zr-based MG shifts to predominantly abrasive wear with only limited oxidative contribution.

### 3.5. Zr-Based MG Subsurface Analysis in Subzero and Room Temperature

**Figure 5:**
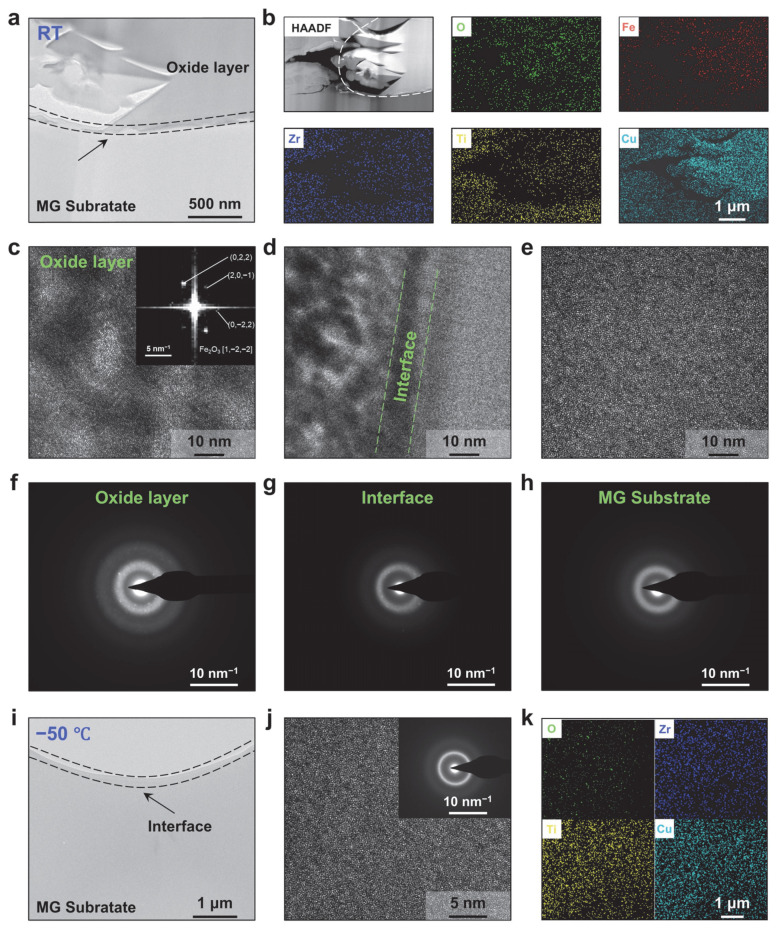
Subsurface analysis of Zr-based MG wear at subzero and room temperatures. (**a**) The cross-sectional image of the subsurface after room-temperature wear. (**b**) High-angle annular dark-field scanning transmission electron microscopy (HAADF-STEM) image and EDS elemental maps of the corresponding region. HRTEM images of (**c**) the oxide layer, (**d**) the bonding interface and (**e**) the substrate. Selected-area electron diffraction (SAED) patterns of (**f**) the oxide layer, (**g**) the bonding interface and (**h**) the substrate. (**i**) The cross-sectional image of the subsurface after subzero wear. (**j**) HRTEM images of the subsurface (the corresponding SAED pattern is shown in the insert) and (**k**) corresponding EDS mapping.

To further elucidate the chemical composition of the oxide layer, detailed characterizations of the subsurface regions after wear at room temperature and −50 °C were performed. Figure 5a presents a cross-sectional image of the subsurface after room-temperature wear, clearly showing a tightly bonded oxide layer atop the Zr-based MG substrate, corroborating the occurrence of adhesive wear [48,49]. Figure 5b displays EDS elemental maps of this region, revealing dense distributions of O and Fe within the oxide layer alongside detectable Zr, Ti and Cu from the MG substrate; conversely, the substrate region itself is dominated by Zr, Ti and Cu, with much lower O and Fe concentrations. Figure 5c–e show HRTEM images of the oxide layer, the bonding interface and the substrate, respectively. In the oxide layer, nanocrystals embedded within the amorphous matrix are evident, and the SAED pattern exhibits crystalline spots superimposed on the diffuse amorphous ring (Figure 5f), indicating the coexistence of crystalline and amorphous phases. FFT analysis combined with EDS data identifies these nanocrystals as Fe_2_O_3_, with diffraction rings corresponding to the (022), (20$\bar{1}$) and (0$\bar{2}$2) reflections. Notably, although the presence of crystalline oxides can enhance hardness (~15–18 GPa) and brittleness [50,51]; under severe abrasion, these hard, brittle oxide fragments fracture into fine wear particles, accelerating the overall wear of Zr-based MG. In the bonding interface region, small crystallites are also observed, though the predominant structure remains amorphous (Figure 5d,g), while the substrate region exhibits only a bright amorphous diffraction ring, confirming the absence of crystalline phases (Figure 5e).

Additionally, the subsurface region after cryogenic wear was examined. Compared to the room-temperature subsurface, the −50 °C subsurface exhibits a markedly smoother morphology with no evidence of oxide layer formation (Figure 5i), indicating the absence of adhesive wear under cryogenic conditions. HRTEM images reveal that the amorphous matrix remains uniformly disordered, as further confirmed by the corresponding diffuse SAED ring, with no crystalline phases detected (Figure 5j). Elemental mapping of the corresponding region shows no detectable Fe and only trace O, demonstrating that both adhesive and oxidative wear products are negligible at −50 °C (Figure 5k).

### 3.6. Zr-Based MG Mechanical Properties in Subzero Temperature

**Figure 6:**
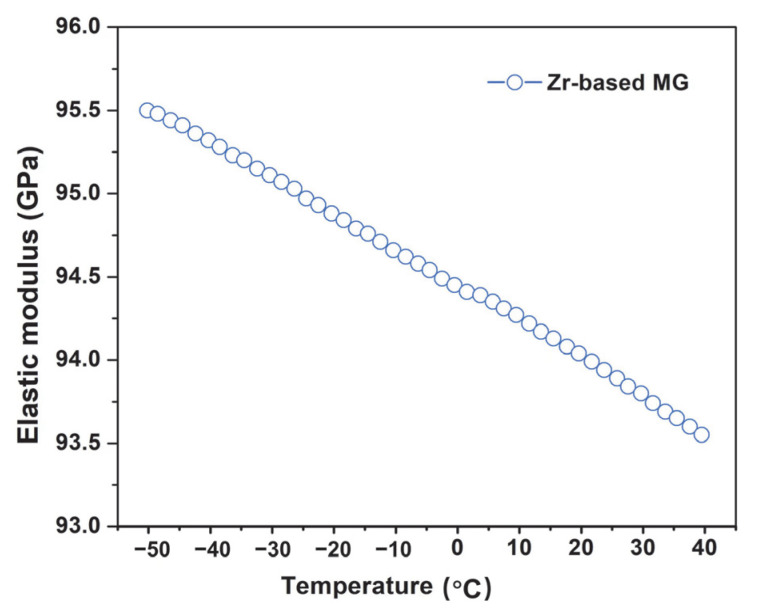
The relationship between the elastic modulus and the temperature of Zr-based MG.

The mechanical properties of the Zr-based MG under low temperature conditions were also evaluated. As shown in Figure 6, the elastic modulus exhibited an approximately linear increase as the temperature decreased from room temperature to −50 °C, reaching a maximum value of 95.5 GPa at the lowest temperature. This trend is primarily attributed to the strengthening of atomic bond interactions [52,53]: at lower temperatures, the thermal energy of atomic vibrations decreases, reducing the amplitude of atomic displacements from equilibrium positions. This results in tighter atomic bonding and, consequently, an increase in the elastic modulus [54].

Moreover, the enhanced elastic modulus at low temperatures has a significant influence on friction and wear behavior. According to Hertzian contact theory [55,56,57], an increase in elastic modulus leads to a reduction in the real contact area, thereby decreasing local strain and plastic deformation during sliding. This mitigates wear, thereby improving the wear resistance of the material. These findings suggest that the elevated elastic modulus is one of the key factors enabling the superior wear performance of Zr-based MG under extreme low-temperature conditions.

Notably, this mechanism differs from the cryogenic wear behavior of crystalline alloys reported in [47]. CoCrFeMnNi high-entropy alloy (HEA) at −50 °C exhibited a wear rate reduction attributed to increased work hardening and suppressed oxidative wear, whereas our Zr-based MG shows dominant abrasive wear with minimal oxidation. The HEA’s crystalline structure promotes dislocation-mediated hardening, while the MG’s amorphous structure relies on elastic modulus enhancement (12% increase at −50 °C) to reduce contact deformation [34,35]. This contrast highlights the unique tribological advantages of amorphous alloys at subzero temperatures, where their disordered structure avoids crystalline defect-induced failure modes.

## 4. Conclusions

In summary, this study conducted low-temperature wear tests on Zr-based MG and systematically analyzed its wear mechanisms and subsurface characteristics. Compared to room temperature, the Zr-based MG exhibited a significantly reduced wear rate and coefficient of friction at −50 °C, with the wear rate reaching approximately one-sixth of that at room temperature. This substantial improvement in wear performance is primarily attributed to the distinct wear mechanisms and mechanical behavior of the Zr-based MG under cryogenic conditions. At low temperatures, the predominant wear mechanism is abrasive wear, with a minor contribution from oxidative wear. In contrast, at room temperature, the rapid sliding contact between the counterface and the material surface generates high localized temperatures, promoting the formation of a hard and brittle oxide layer at the interface. This, in turn, leads to severe oxidative wear, adhesive wear and abrasive wear through a synergistic mechanism. Thus, temperature plays a critical role in governing the wear behavior of Zr-based MGs. Cryogenic environments effectively suppress intense oxidation and adhesion, enhancing the overall wear resistance of Zr-based MG. This work not only broadens the potential mechanical applications of MGs in cryogenic conditions but also offers a promising material candidate for equipment development in fields such as deep space exploration and polar research.

## Figures and Tables

**Table 1:** Distribution of elements in wear region under subzero and room temperature.

	O (at.%)	Fe (at.%)	Zr (at.%)	Ti (at.%)	Cu (at.%)
Spot 1	50.41	10.35	15.00	18.55	5.7
Spot 2	8.16	1.14	35.17	41.37	14.16
Spot 3	25.78	2.79	29.44	32.06	9.93
Spot 4	7.7	0.62	36.04	41.77	13.87

## Data Availability

The original contributions presented in this study are included in the article. Further inquiries can be directed to the corresponding authors.
